# Supplementary material for: Genetic Background Influences Severity of Colonic Aganglionosis and Response to GDNF Enemas in the Holstein Mouse Model of Hirschsprung Disease
Source: Int J Mol Sci. 2021 Dec 5;22(23):13140. doi: 10.3390/ijms222313140 (PMC8658428; doi:10.3390/ijms222313140)
Supplement: Supplementary file 1 [file ijms-22-13140-s001.zip › Figure S1.pdf]

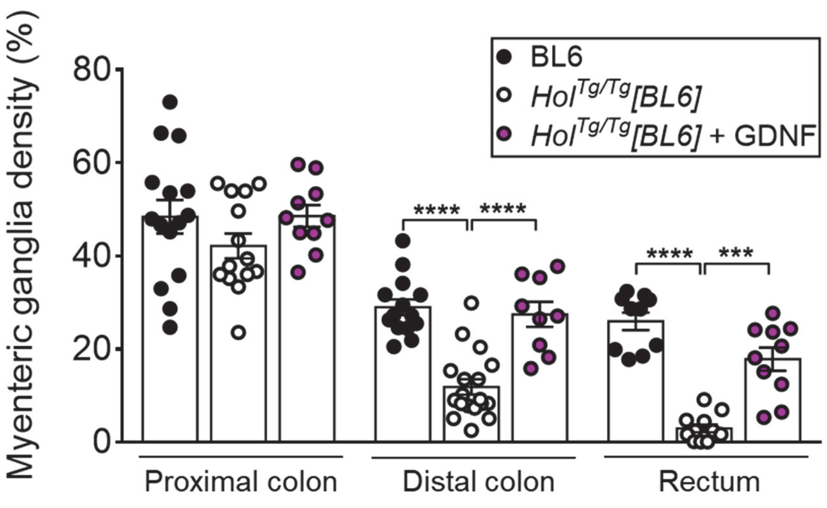

Figure S1. Quantitative analysis of GDNF-induced neurogenesis in the colon of P20 *Hol*$^{Tg/Tg}$*[BL6]* mice. Quantification of the surface area covered by HuC/D+ myenteric ganglia in P20 *Hol*$^{Tg/Tg}$*[BL6]* mice that were administered GDNF enemas between P4-P8, using images such as those displayed in Figure 5a. Each value is a field a view, for a minimum of 3 fields of view per animal (n=3-5 animals per group). ****P<0.0001, two-way ANOVA with post-hoc Sidak's test.
