# Supplementary material for: Genetic Background Influences Severity of Colonic Aganglionosis and Response to GDNF Enemas in the Holstein Mouse Model of Hirschsprung Disease
Source: Int J Mol Sci. 2021 Dec 5;22(23):13140. doi: 10.3390/ijms222313140 (PMC8658428; doi:10.3390/ijms222313140)
Supplement: Supplementary file 1 [file ijms-22-13140-s001.zip › Figure S2.pdf]

a

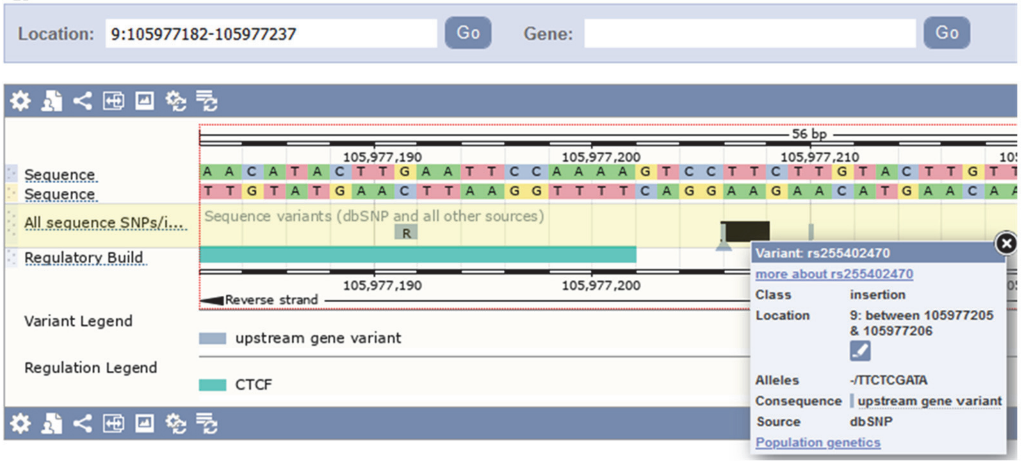

b

```
C57BL/6N    1 atttacaacggctggctcacagtattctctcaataaatattcccagaata   50
              ||||||||||||||||||||||||||||||||||||||||||||||||||
   FVB/N    1 atttacaacggctggctcacagtattctctcaataaatattcccagaata   50

C57BL/6N   51 aagaggtgaacagatattcacctcagaaacatacttgaattccaaaagtc  100
              |||||||||||||||||||||||||||||||||||.||||||||||||||
   FVB/N   51 aagaggtgaacagatattcacctcagaaacatacttaaattccaaaagtc  100

C57BL/6N  101 c---------ttcttgtacttgttaggacattgccaccttcctggtcatg  141
              |         ||||||||||||||||||||||||||||||||.||||||
   FVB/N  101 cttctcgatattcttgtacttgttaggacattgccaccttcctagtcatg  150

C57BL/6N  142 acacttcctggtgtcatgctatgg  165
              ||||||||||||||||||||||||
   FVB/N  151 acacttcctggtgtcatgctatgg  174
```

Figure S2. The CTCF-enriched region upstream of *Col6a4* varies as a function of genetic background. (**a**) Zoom-in view of the intergenic region between *Col6a4* and *Glyctk* on the Ensembl Genome Browser (useast.ensembl.org), showing the position of a 12-bp indel variant next to a ChIP-seq peak of CTCF binding. (**b**) This 12-bp sequence is present in the FVB/N background but not in the C57BL/6 background, as indicated here by sequence comparison of this region amplified from $Hol^{Tg/Tg}$*[BL6]* and $Hol^{Tg/Tg}$*[FVB]* mice.
