# Supplementary material for: Genetic Background Influences Severity of Colonic Aganglionosis and Response to GDNF Enemas in the Holstein Mouse Model of Hirschsprung Disease
Source: Int J Mol Sci. 2021 Dec 5;22(23):13140. doi: 10.3390/ijms222313140 (PMC8658428; doi:10.3390/ijms222313140)
Supplement: Supplementary file 1 [file ijms-22-13140-s001.zip › Table S1.pdf]

Table S1. Antibodies used in this study.

Antibody	Dilution	Host species	Source
βIII-Tubulin	1:500	Mouse	Abcam, ab78078
Collagen VI	1:500	Rabbit	Abcam, ab6588
HuC/HuD	1 :500	Mouse	Molecular Probes, A-21271
Anti-rabbit Alexa Fluor 594	1 :500	Donkey	Jackson ImmunoResearch Laboratories Inc, 711-585-152
Anti-mouse Alexa Fluor 647	1 :500	Donkey	Jackson ImmunoResearch Laboratories Inc, 715-605-150
