# Supplementary material for: Genetic Background Influences Severity of Colonic Aganglionosis and Response to GDNF Enemas in the Holstein Mouse Model of Hirschsprung Disease
Source: Int J Mol Sci. 2021 Dec 5;22(23):13140. doi: 10.3390/ijms222313140 (PMC8658428; doi:10.3390/ijms222313140)
Supplement: Supplementary file 1 [file ijms-22-13140-s001.zip › Table S2.pdf]

Table S2. Oligonucleotide primers used in this study.

Gene	Sense primer	Antisense primer
**RT-qPCR**		
Col6a1	5’-GAGGATAACAACGACATTTCACCC	5’-TGACCTTGATGATGAAGTCCTTGG
Col6a2	5’-AGATTTGGTCTGAAAGGAACACC	5’-ATACTCTCAGAACTGTCGATGACG
Col6a4	5’-AAGAGGATTTTCAGGAGAGAAGGG	5’-AGATTATCAATTCCAGGATCCCCC
Gapdh	5′-TCCTGCACCACCAACTGCTTAGC	5′-AGGTCCACCACCCTGTTGCTGTA
**Genotyping**		
Holstein F	5’- GTGGTGGACCTAACCTTACAAGGA	--
Holstein R1	--	5’-CAGGGCTAAGTCTTGGCTTACTTG
Holstein R2	--	5’-CACAGCTTGCTGTATCAGAGCCAT
G4-RFP	5′-GGGCTGTCATCTCACTATGGGCA	5′-TGATTATGTCCCCATGACTGTCAG
